# Diagnostic Performance of a Novel Noninvasive Workup in the Setting of Dry Eye Disease

**DOI:** 10.1155/2020/5804123

**Published:** 2020-12-11

**Authors:** Luca Vigo, Marco Pellegrini, Federico Bernabei, Francesco Carones, Vincenzo Scorcia, Giuseppe Giannaccare

**Affiliations:** ^1^Carones Ophthalmology Center, Milan 20122, Italy; ^2^Ophthalmology Unit, S.Orsola-Malpighi University Hospital, University of Bologna, Bologna 40138, Italy; ^3^Department of Ophthalmology, University Magna Græcia of Catanzaro, Catanzaro 88100, Italy

## Abstract

**Purpose:**

To evaluate the diagnostic performance of a novel noninvasive automated workup employed for the diagnosis of dry eye disease (DED).

**Methods:**

One hundred patients with mild to moderate DED and 100 matched control subjects were enrolled in this cross-sectional study. Ocular surface examinations were carried out by means of IDRA Plus (SBM Sistemi, Turin, Italy), which allows the automated evaluation of noninvasive breakup time (NIBUT), lipid layer thickness (LLT), tear meniscus height (TMH), infrared meibography for the measurement of meibomian gland loss (MGL), and blinking analysis. Continuous variables were compared between patients with DED and controls by using the Mann–Whitney *U* test. The area under the curve (AUC) of receiver operating characteristic curves was calculated. The correlations between ocular surface parameters were evaluated with Pearson correlation analysis.

**Results:**

Patients with DED showed significantly lower values of NIBUT, LLT, and TMH compared to controls (6.9 ± 2.5 vs 10.4 ± 2.4 s, *P* < 0.001; 64.6 ± 20.3 vs 73.4 ± 21.9 nm, *P* = 0.003; 0.231 ± 0.115 vs 0.289 ± 0.164, *P* = 0.012, respectively). Conversely, no significant differences were observed for MGL and blinking analysis (both *P* > 0.05). NIBUT had the highest diagnostic power (AUC = 0.841, sensitivity = 0.89, and specificity = 0.69), followed by LLT (AUC = 0.621, sensitivity = 0.89, and specificity = 0.55), TMH (AUC = 0.606, sensitivity = 0.57, and specificity = 0.63), blink analysis (AUC = 0.533, sensitivity = 0.48, and specificity = 0.59), and MGL (AUC = 0.531, sensitivity = 0.54, and specificity = 0.48). In patients with DED, NIBUT showed a significant correlation with TMH (*R* = 0.347, *P* = 0.002) and blinking analysis (*R* = 0.356, *P* < 0.001), while blinking analysis was negatively correlated with MGL (*R* = −0.315, *P* = 0.008).

**Conclusions:**

The automated noninvasive workup validated in this study may be a useful tool for reaching a noninvasive diagnosis of DED with a good performance, especially for NIBUT.

## 1. Introduction

Dry eye disease (DED) is a multifactorial disease of tears and ocular surface that represents one of the most frequent ophthalmological complaints, affecting hundreds of millions of people worldwide [1]. Based on the definition by Tear Film and Ocular Surface Society Dry Eye Workshop (TFOS DEWS) II, multiple factors including tear film instability, tear hyperosmolarity, inflammation, and neurosensory abnormalities play a key role in the pathogenesis of DED [2].

No single gold-standard diagnostic marker has yet been established, mainly due to different aspects of the disease including the multifactorial and complex pathogenesis, the poor correlation between symptoms and signs, and the significant fluctuation over time and season of currently available metrics [3–6]. As such, nowadays the diagnosis of DED is reached if ocular discomfort symptoms are present in association with at least one marker of disrupted homeostasis of the ocular surface among corneal staining, tear film instability, and increased tear osmolarity [7].

Recently, novel metrics, including among others tear film interferometry, infrared meibography, tear meniscus height, and evaluation of blinking characteristics [8–14], have been developed to complement the diagnosis of DED traditionally reached with slit-lamp examination and vital dye staining. The advantages of these tests include the noninvasive nature and the automated calculation of the results that can provide reliable biomarkers of the disease, avoiding observer bias [15]. However, the current lack of validated cutoff values for reaching the diagnosis of DED hampered their wide adoption in the clinical practice.

The purpose of this study was to evaluate the performance of a novel noninvasive automated workup for reaching the diagnosis of DED.

## 2. Materials and Methods

### 2.1. Study Design

This cross-sectional study was conducted at the Department of Ophthalmology of the University Magna Græcia of Catanzaro (Italy) between December 2019 and February 2020. The study was performed in accordance with the principles of the Declaration of Helsinki and was approved by the local ethics committee (Comitato Regione Calabria Sezione Area Centro-Protocol n. 280/2019). Consecutive patients over 18 years of age with a confirmed diagnosis of DED who attended the ocular surface office for control visits were screened for enrolment. The diagnosis of DED was reached according to TFOS DEWS II criteria, which require an ocular surface disease index score ≥ 13 plus one between tear breakup time (TBUT) < 10 seconds or > 5 spots of corneal staining [7]. Healthy subjects attending our center for routine ophthalmic visits, who were matched by age and gender, were selected as the control group. Exclusion criteria for both groups were contact lens wearing, previous corneal surgery, and active ocular diseases including allergy as well as uncontrolled systemic diseases.

### 2.2. Ocular Surface Examination

**Figure 1:**
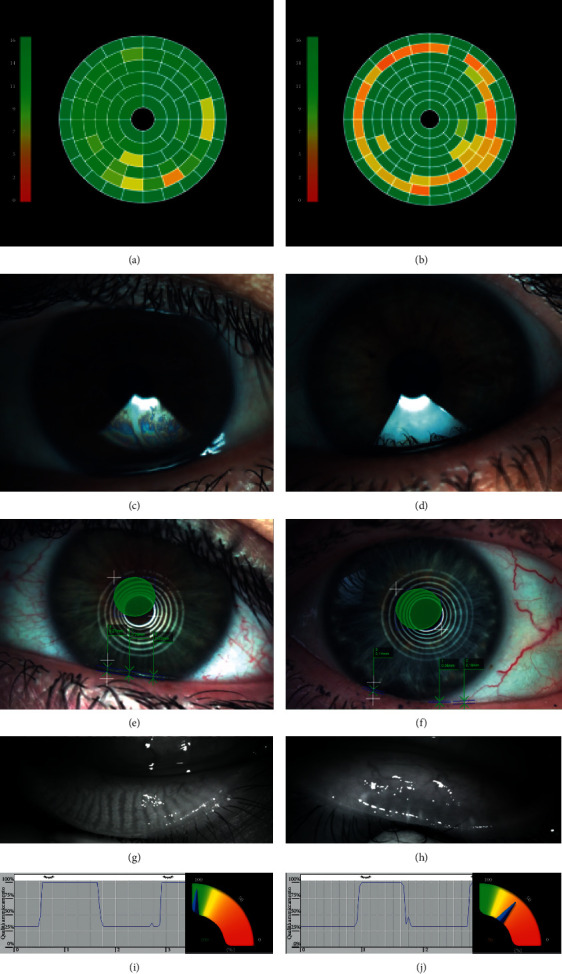
Ocular surface workup in a representative control subject (a, c, e, g, i) and in a patient with dry eye disease (b, d, f, h, j). (a, b) Measurement of noninvasive breakup time. (c, d) Tear film interferometry for the measurement of lipid layer thickness. (e, f) Measurement of tear meniscus height. (g, h) Infrared meibography. (i, j) Blink analysis.

All ocular surface examinations were performed using the newly developed IDRA Plus (SBM Sistemi, Turin, Italy), an all-in-one device which allows the automated measurement of (i) noninvasive breakup time (NIBUT) (Figures 1(a) and 1(b)); (ii) lipid layer thickness (Figures 1(c) and 1(d)); (iii) tear meniscus height (Figures 1(e) and 1(f)); (iv) infrared meibography (Figures 1(g) and 1(h)); and (v) blinking analysis (Figures 1(i) and 1(j)). In detail, NIBUT was measured without the need for fluorescein dye after asking the patient to blink 3 consecutive times and then hold the eyes open. The measurement was repeated 3 times, and the mean value was recorded. Lipid layer thickness was estimated by observing the interference pattern and colours of the moving lipid tear film. Tear meniscus height was measured along the lower lid margin immediately below the pupil. Infrared meibography was performed after everting the superior eyelid, and meibomian gland loss was defined as the percentage of gland loss in relation to the total tarsal area of the lid. The blinking analysis was performed by recording a 30-second video while the patient was asked to blink naturally by avoiding forced blinking, and the percentage closure of maximal palpebral fissure opening was noted. The tests were performed in the following chronology in order to avoid/minimize potential confounding effects on the readings of subsequent measurements [16]: blink analysis, tear meniscus height, lipid layer thickness, NIBUT, and infrared meibography.

### 2.3. Statistical Analysis

The statistical analysis was conducted with R (version 4.0.0) and RStudio (version 1.2.5042) software. Examinations were performed in both eyes of patients, and values from the worst eye according to TFOS DEWS II criteria were used for the statistical analysis. Continuous variables were compared between patients with DED and controls by using the Mann–Whitney *U* test. Receiver operating characteristic (ROC) curves were drawn to assess the diagnostic significance of ocular surface parameters by using the pROC package [17]. The accuracy of each ocular surface parameter for discriminating patients with DED from controls was evaluated by calculating the area under the curve (AUC). The optimal cutoff value of each parameter was determined as the point on the ROC curve that was nearest to the coordinate (1, 1). The correlations between ocular surface parameters were evaluated with Pearson correlation analysis. A Bonferroni correction for multiple comparisons was applied. A *P* value < 0.05 was considered statistically significant.

## 3. Results

Overall, 100 eyes of DED patients and 100 eyes of control subjects were included. No significant differences between the two groups were observed for gender distribution (74% females in the DED group vs 70% females in the control group, *P* = 0.637) and age (50.5 ± 31.1 years vs 54.0 ± 14.7, *P* = 0.075).

The results of the ocular surface examination in the two groups are reported in Table 1. Compared to control subjects, patients with DED showed a significantly lower NIBUT (*P* < 0.001), lipid layer thickness (*P* = 0.003), and tear meniscus height (*P* = 0.012). Conversely, no significant differences in meibomian gland loss and blinking analysis were observed (both *P* > 0.05).

**Figure 2:**
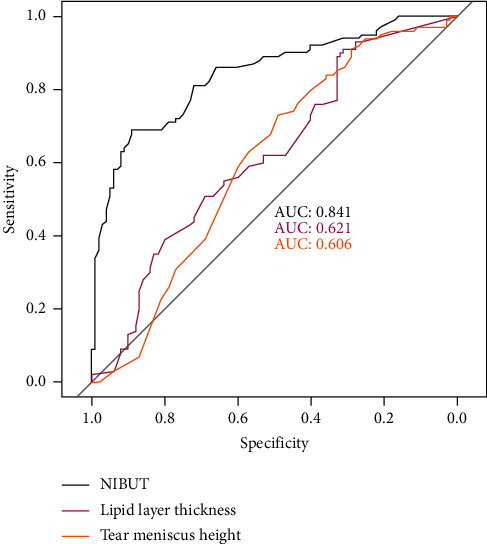
Receiver operating characteristic curves with area under the curve of noninvasive breakup time, lipid layer thickness, and tear meniscus height for the diagnosis of dry eye disease.

The AUC of ROC curves along with optimal cutoff values with corresponding sensitivity and specificity of the ocular surface parameters analyzed is reported in Table 2: NIBUT had the highest diagnostic power (AUC = 0.841, sensitivity = 0.89, and specificity = 0.69), followed by lipid layer thickness (AUC = 0.621, sensitivity = 0.89, and specificity = 0.55), tear meniscus height (AUC = 0.606, sensitivity = 0.57, and specificity = 0.63), blinking analysis (AUC = 0.533, sensitivity = 0.48, and specificity = 0.59), and meibomian gland loss (AUC = 0.531, sensitivity = 0.54, and specificity = 0.48).  Figure 2 shows the ROC curves of NIBUT, lipid layer thickness, and tear meniscus height.

In patients with DED, NIBUT showed a significant correlation with tear meniscus height (*R* = 0.347, *P* = 0.002) and blinking analysis (*R* = 0.356, *P* < 0.001); moreover, blinking analysis was negatively correlated with meibomian gland loss (*R* = −0.315, *P* = 0.008). No other significant correlations were observed.

## 4. Discussion

The prevalence of DED varies consistently across different population, and this is partially due to the heterogeneity of diagnostic criteria used in different studies [1]. To address this issue, the TFOS DEWS II guidelines developed a consensus diagnostic battery of tests for DED including breakup time, tear osmolarity, ocular surface staining, and symptomatology [7]. Nevertheless, the DEWS II Diagnostic Methodology Subcommittee acknowledged the lack of a gold-standard test to diagnose DED and the need of identifying new reliable biomarkers [7]. In the same report, it has been highlighted that studies evaluating novel diagnostic tests are frequently affected by selection and spectrum biases. The former occurs when a novel test is compared to established ones that were used as inclusion criteria, resulting in apparently poor performance. The latter refers to the exclusion from clinical trials of patients with mild disease, with overestimation of the diagnostic performance. Conversely, to avoid both these biases and obtain reliable estimates of the diagnostic performance, novel tests should be developed and validated using data from the population in which they are intended to be used [18]. Therefore, in the present study, we included consecutive patients with a confirmed diagnosis of DED presenting to our center for routine control visits. Since DED diagnosis had been already reached previously, we did not use conventional tests to select and grade patients. This resulted in the inclusion of a broad population of mild to moderate DED patients, producing results that are generalizable to real-life clinical practice.

Patients with DED showed significantly lower values of NIBUT, lipid layer thickness, and tear meniscus height compared to controls, while no differences in meibomian gland loss and blinking analysis were observed. The ROC analysis showed that NIBUT was the parameter with the highest sensitivity and specificity to diagnose DED. Lipid layer thickness and tear meniscus height had moderate diagnostic utility, while the performances of meibomian gland loss and blinking analysis were poor.

In agreement with these results, also previous works focused on both hyposecretory [19] and evaporative DED [10] found that NIBUT was the best single diagnostic test for reaching the diagnosis. Our results further confirm the role of tear film instability measurement as a reliable indicator of DED diagnosis. Compared to conventional breakup time, NIBUT has the advantage of avoiding contact with the ocular surface as well as disruption of the tear film induced by fluorescein instillation [20]. Interestingly, the optimal NIBUT cutoff value in this study was 7.75 seconds, which is lower than the cutoff of 10 seconds proposed in previous works [7, 21].

Tear meniscus height and lipid layer thickness showed moderate diagnostic performance to differentiate DED from controls. Singh and colleagues recently reported higher accuracy of tear meniscus height (sensitivity 0.98 and specificity 0.96) in patients with moderate to severe DED [13]. Not surprisingly, the performance of this parameter in patients with milder diseases, like those included in our study, was found to be lower. Conversely, the previously reported accuracy of lipid layer thickness for the diagnosis of meibomian gland dysfunction (sensitivity 0.65 and specificity 0.63) is consistent with the results of our study [22].

No differences in meibography between DED and controls were observed in this study. Although we did not classify patients according to the subtype of DED (aqueous, evaporative, or mixed), this finding could be explained by the limited number of patients with evaporative DED included in the study. In fact, it has been shown that meibomian gland changes are usually more pronounced in meibomian gland dysfunction compared to DED of other types [23].

Although blinking analysis showed limited diagnostic utility, this parameter showed a significant but relatively weak correlation with both NIBUT and meibomian gland loss. Jie and coauthors reported similar associations and speculated that incomplete blinking could lead to inadequate meibomian gland expression and subsequent tear film instability [14]. It should be noted that a standardized methodology to evaluate incomplete blinking has not yet been developed. We measured the percentage of eye closure while patients blinking spontaneously, but also other methods such as the incomplete blink rate might provide additional information for the characterization of eyelid dynamics [24, 25].

This study suffers from some limitations that deserve mentioning. In particular, in order to best reflect everyday practice, we included patients with a confirmed diagnosis of DED regardless of disease severity and/or DED subtype. Further studies with more rigorous inclusion criteria are needed to evaluate the possible changes of diagnostic performance in different DED scenarios. Furthermore, future studies are warranted to investigate the correlation between the results obtained with this new noninvasive diagnostic device and DED clinical and molecular findings.

## 5. Conclusions

The automated noninvasive workup presented and validated in this study may be a useful tool to diagnose DED with good values of sensitivity and specificity, especially for NIBUT. Furthermore, since the effects of this workup on volume or properties of the tear film are negligible, it can be used as an effective screening tool for discriminating healthy subjects from patients affected or at risk for DED, before proceeding with invasive ocular surface examinations required for a better characterization of the disease.

## Figures and Tables

**Table 1:** Ocular surface parameters in patients with dry eye disease and control subjects.

Parameter	Dry eye group (*n* = 100)	Control group (*n* = 100)	*P* value
NIBUT (s)	6.9 ± 2.5	10.4 ± 2.4	**<0.001**
Lipid layer thickness (nm)	64.6 ± 20.3	73.4 ± 21.9	**0.003**
Tear meniscus height (mm)	0.231 ± 0.115	0.289 ± 0.164	**0.012**
Meibomian gland loss (%)	22.4 ± 12.9	20.3 ± 11.4	0.458
Blink analysis	85.0 ± 19.5	87.2 ± 18.8	0.382

**Table 2:** Area under the curve (AUC) with 95% confidence intervals (CIs), optimal cutoff values, and corresponding sensitivity and specificity for the analyzed ocular surface parameters.

Parameter	AUC	95% CI	Cutoff	Sensitivity	Specificity
NIBUT (s)	0.841	0.786–0.895	7.75	0.89	0.69
Lipid layer thickness (nm)	0.621	0.543–0.699	66.5	0.64	0.55
Tear meniscus height (mm)	0.606	0.527–0.685	0.225	0.57	0.63
Meibomian gland loss (%)	0.531	0.450–0.611	17.5	0.54	0.48
Blink analysis	0.533	0.460–0.606	99.0	0.48	0.59

## Data Availability

The data that support the findings of this study are available from the corresponding author upon reasonable request.
